# Genetic variants in BAT2 are associated with immune responsiveness to influenza vaccination

**DOI:** 10.3389/fgene.2023.1059447

**Published:** 2023-02-09

**Authors:** Simin Wen, Hejiang Wei, Mao Li, Shuyi Zhong, Yanhui Cheng, Weijuan Huang, Dayan Wang, Yuelong Shu

**Affiliations:** ^1^ Guangzhou First People’s Hospital, The Second Affiliated Hospital of South China University of Technology, Guangzhou, China; ^2^ National Institute for Viral Disease Control and Prevention, Chinese Center for Disease Prevention and Control, Beijing, China; ^3^ School of Public Health (Shenzhen), Sun Yat-sen University, Shenzhen, China; ^4^ Institute of Pathogen Biology, Chinese Academy of Medical Sciences and Peking Union Medical School, Beijing, China

**Keywords:** influenza, vaccine, immune response, BAT2, single nucleotide polymorphism

## Abstract

**Background:** Influenza is a global public health problem for its detrimental impact on human health. Annual vaccination is the most effective prevention of influenza infection. Identifying host genetic factors associated with the responsiveness to influenza vaccines can provide clues for developing more effective influenza vaccines. In this study, we aimed to explore whether the single nucleotide polymorphisms in BAT2 are associated with the antibody responses to influenza vaccines.

**Method:** A nested case-control study was conducted in this research. 1968 healthy volunteers were enrolled and 1,582 of them from a Chinese Han population were eligible for further research. According to the hemagglutination inhibition titers of subjects against all influenza vaccine strains, a total of 227 low responders and 365 responders were included in the analysis. Six tag single nucleotide polymorphisms in the coding region of BAT2 were selected and genotyped using the MassARRAY technology platform. Univariable and multivariable analyses were conducted to evaluate the relationship between variants and antibody responses to influenza vaccination.

**Results:** Multivariable logistic regression analysis showed that, compared with the *BAT2* rs1046089GG genotype, the GA + AA genotype was correlated with decreased risk of low responsiveness to influenza vaccines after adjusting for gender and age (*p* = 1.12E-03, OR = .562, 95%CI: .398–.795). rs9366785 GA + AA genotype was associated with a higher risk of low responsiveness to influenza vaccination compared with the GG genotype (*p* = .003, OR = 1.854, 95%CI: 1.229–2.799). The haplotype consisting of *BAT2* rs2280801-rs10885-rs1046089-rs2736158-rs1046080-rs9366785 CCAGAG was correlated with a higher level of antibody response to influenza vaccines compared with haplotype CCGGAG (*p* < .001, OR = .37, 95%CI: .23–.58).

**Conclusion:** Genetic variants in BAT2 were statistically associated with the immune response to influenza vaccination among the Chinese population. Identifying these variants will provide clues for further research on novel broad-spectrum influenza vaccines, and improve the individualized influenza vaccination scheme.

## Introduction

Influenza is an acute viral respiratory disease that has caused a substantial disease burden worldwide (Uyeki et al., 2022). A systematic review and meta-analysis estimated that approximately 32.13 million influenza-associated lower respiratory infections and 5.68 million hospitalizations occur worldwide each year. It is also reported that more than 14% of acute respiratory hospitalizations among adults were influenza-related cases (Lafond et al., 2021). The severity of influenza infection varies widely. Some vulnerable people including pregnant women, young children, the elderly and those with underlying conditions may experience severe or fatal complications after the infection. The best prevention of influenza is primarily by annual vaccination. To develop new influenza vaccines with improved effectiveness, factors that may influence the responsiveness to vaccines should be fully identified.

Genetic variants in immune-related genes have an important impact on the immune response to vaccines. Human leukocyte antigen (HLA) is a polygenic and polymorphic complex that plays a key role in the process of antigen presentation. Up to now, a series of studies have reported that some single nucleotide polymorphisms (SNPs) in HLA regions may be related to the responsiveness to influenza vaccines (Wen et al., 2021). These reported genes are primarily located in the HLA class I and II region. No association studies have been conducted for genes in the HLA class III region. The class III region of the HLA, which has very high gene density, lies between regions I and II, and contains a cluster of genes that have certain functions in immunology and inflammation (Xie et al., 2003).

HLA-B associated transcript 2 (BAT2), also known as PRRC2A, is located in the HLA class III region (6p21.33) and also in the vicinity of the genes for tumor necrosis factor (TNF) alpha and TNF beta. BAT2 encodes a large number of proline-rich proteins and is divided into three fragments corresponding to the amino acids 1–773, 756–1,408, or 1,391–2,157. Previous studies on this gene mostly focused on the relationship between genetic variants in BAT2 and susceptibility to various diseases. Only a few studies have been conducted to reveal the specific function of this gene. A study using high-throughput protein-interaction assays showed that BAT2 is a kind of RNA-binding protein that plays a role in regulating pre-mRNA splicing (Schott and Garcia-Blanco, 2021). The N terminus of BAT2 interacts with proteins involved in mRNA processing, including heterogeneous nuclear ribonucleoprotein (hnRNP) A1 and hnRNP M, both of which are components of the spliceosome. The C-terminal fragment of BAT2 interacted promiscuously with many different proteins, whereas all of the colonies obtained with the middle fragment of BAT2 corresponded to a single interacting protein, C1QBP, which interacts with the alternative splicing factor/splicing factor 2 (ASF/SF2) (Lehner et al., 2004). A recent study reported that BAT2 is a reader for the common epitranscriptome mark N^6^-methyladenosine (m^6^A) in mRNAs and plays an important role in oligodendrocyte progenitor cells proliferation and oligodendrocyte fate determination. Transcriptome-wide analysis showed that Olig2 is a critical downstream target gene of BAT2 in oligodendrocyte development. BAT2 stabilizes Olig2 mRNA through binding to a consensus GGACU motif in the Olig2 coding sequence in an m6A-dependent manner (Wu et al., 2019). While many researchers have confirmed the influence of BAT2 on the immune system through genetic association studies, no research has revealed its specific role in it. Based on the location of BAT2, some researchers speculated that the immunological effect of this gene may due to the linkage-disequilibrium (LD) between BAT2 and loci in the region of HLA class I, HLA class II and TNF (Fugger et al., 1990), but no confirmation has yet been made.

A variety of studies have reported that the genetic variants in BAT2 were associated with susceptibility to numerous immune-mediated diseases, such as rheumatoid arthritis (Singal et al., 2000), ulcerative colitis (Fernández et al., 2005), Kawasaki disease (Hsieh et al., 2010), non-Hodgkin lymphoma (Nieters et al., 2012) and multiple sclerosis (J. Zhang et al., 2021). It has been reported that the mutation in BAT2 was correlated with the antibody levels of Pseudorabies virus in pigs (Wang et al., 2012). However, the association of genetic variants in BAT2 with the immune response to vaccines in humans remains unexplored.

In this study, to investigate associations between SNPs in BAT2 and the responsiveness to influenza vaccination, we selected six tag SNPs in the coding region of BAT2 and detected them in 592 subjects of Chinese descent. The identification of these associated variants may provide clues for the research and development of novel broad-spectrum influenza vaccines, improve the individualized influenza vaccination scheme, and also help to further understand the function of BAT2 in the immune system.

## Materials and methods

### Study population

A nested case-control study was conducted in this research. Initially, from September 2009 to September 2019, 1968 healthy volunteers with informed consent were enrolled from Urumqi Center for Disease Control and Prevention (CDC) and Yunnan CDC. This study was reviewed and approved by the Ethics Review Committee of the National Institute for Viral Disease Control and Prevention (NIVDC, assurance number, 200916). Subjects aged over 5 years old were inoculated with a trivalent inactivated vaccine (TIV) containing 15 μg hemagglutinin (HA) in each component. Children under 5 years of age received two doses of TIV, each containing 7.5 μg HA, with an interval of 30 days. For subjects recruited in the same year, they received the same vaccine strains. In Supplementary Table S1, we summarized the recommended vaccine strains from the World Health Organization (WHO) for the northern hemisphere from 2009 to 2019. The exclusion criteria were as follows: non-Han Chinese origin (*n* = 14); lost to follow-up (*n* = 31); inadequate blood samples (*n* = 210) and subjects with a history of influenza vaccination (*n* = 131). As a result, 386 participants were excluded, and in total, 1,582 subjects were available for further research.

**FIGURE 1:**
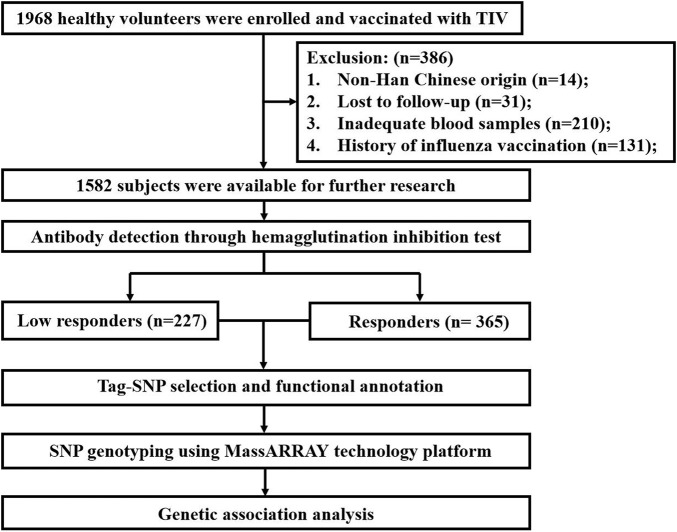
Study flow chart.

In this study, all subjects were divided into four age groups, including infants (<5 years), children (5–17 years), adults (18–64 years) and the elderly (≥65 years). Seroconversion was defined as subjects with either a pre-vaccination hemagglutination inhibition (HI) titer <1:10 and post-vaccination HI titer≥1:40 or a pre-vaccination HI titer≥1:10 and a minimum 4-fold increase in post-vaccination HI titer. Based on the seroconversion levels of subjects against three vaccine strains (H1N1, H3N2 and B), only subjects whose antibody fold rises have achieved or have not achieved the seroconversion to all vaccine strains were included in the analysis. This step reduced the false positive results from analyzing only a single component. Eventually, a total of 592 subjects were selected and divided into two groups: low responders (*n* = 227) and responders (*n* = 365). Low responders were defined as those who have not reached the seroconversion level to any strain. And responders were those who had achieved seroconversion to all components. The screening process of the study participants is shown in Figure 1.

### Test of HI

Blood samples were collected before the inoculation and 28 days after the last inoculation. HI assays were performed according to the standardized protocol by Technical Guidelines for National Influenza Surveillance. Before the test, receptor destroying enzyme (RDE) and red blood cells (RBCs) were mixed with serum respectively to remove the non-specific inhibin and non-specific lectin from serum. Turkey RBCs were used to detect the HI titers against H1N1 and B, while H3N2 HI assays were performed using the guinea pig RBCs. The HI titer was determined to be the highest serum dilution to inhibit HA.

#### Selection of tag SNPs and genotyping

**FIGURE 2:**
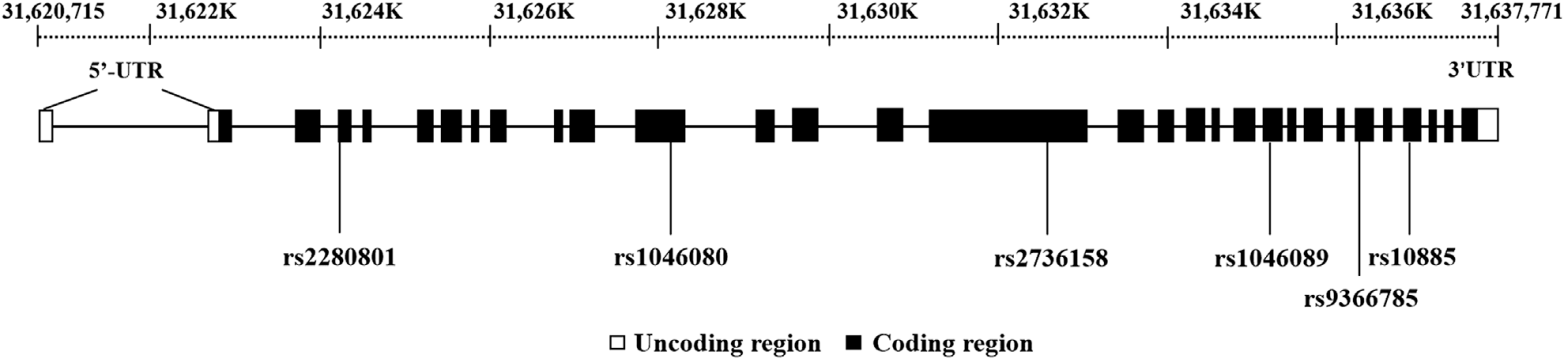
Positions of six tag SNPs on BAT2.

Based on the data from the 1000 Genomes Project Consortium, tag SNPs were selected using Haploview 4.2 program with minor allele frequencies (MAFs) greater than .05 in the Chinese population. The threshold of *r* ^2^ was .8. SNPinfo (https://snpinfo.niehs. nih.gov/) were used for functional annotation. Eventually, six functional tag SNPs in the coding region were selected and included in the analysis. The positions of six tag SNPs on BAT2 were presented in Figure 2.

Genomic DNA was extracted from blood clots using a TIANamp Blood Clot DNA kit (TIANGEN BIOTECH, China) and stored at −80 °C until use. SNPs were genotyped using the MassARRAY technology platform and determined by BioMiao Biological Technology (Beijing, China). SNPs with call rates lower than 95% or Hardy-Weinberg equilibrium (HWE)-*P* less than .001 should be excluded. The optimal genetic model refers to the one with the most statistically significant result.

#### Genetic models

A total of four kinds of genetic models were applied in the analyses. The additive genetic model was used to compare the distribution frequencies of the three genotypes. The other three genetic models divided the subjects into two groups by different combinations of the three genotypes. The dominant genetic model compared the distribution frequencies of wild-type homozygotes with other subjects. The recessive genetic model compared the distribution frequencies of mutant homozygotes with other subjects. The overdominant genetic model divided the subjects into homozygotes and heterozygotes to analyze whether the mutation had heterozygous superiority.

#### Statistical analysis

HWE was assessed using the Chi-square test. Quantitative variables with unnormal distribution and categorical variables were described as median [interquartile range (IQR)] and percentages respectively. The genotypic and allelic frequencies of tag SNPs between groups were compared by Chi-square test or Fisher’s exact test when appropriate. Univariable and multivariable logistic regression analyses adjusted for age and gender were performed to evaluate the associations between different genetic models and the antibody responses to influenza vaccines. HI titers were transformed to their logarithms when calculating the antibody fold rises. Mann-Whitney *U* Test and covariance analysis were conducted to compare the antibody fold rises of different genotype carriers. Linkage disequilibrium was processed by Haploview V.4.2. An online software SNPstats (https://www.snpstats.net/) was used for haplotype analysis. Analyses were performed by IBM SPSS Statistics (version 25.0). *p* < .05 was considered statistically significant. According to the number of SNPs and haplotypes, the Bonferroni correction was used to control for multiple testing (.05/6 = 8.33E-03; .05/12 = 4.17E-03). The minimum sample size was estimated by Quanto software (version 1.2.4) under the statistical power of .80. HaploReg V4.1 online software (https://pubs.broadinstitute.org/mammals/haploreg/haploreg.php) was used for LD information collection. Graphs were processed by GraphPad Prism 8.

## Results

### Characteristics of subjects

A total of 227 low responders and 365 responders were included in the analysis. In Supplementary Table S2, we compared the characteristics of subjects between two groups. As a result, no difference in gender and age distribution was found between low responders and responders (*p* > .05). Compared with infants under 5 years old, children aged 5–17 years old tend to be associated with a higher level of immune response, while the elderly ≥65 years old tend to be correlated with a lower level of antibody response to influenza vaccines, but the differences are not statistically significant.

### Association between tag SNPs and immune response to influenza vaccination

The genotypic and allelic frequencies of six tag SNPs of BAT2 between low responders and responders were summarized in Table 1. The call rates of *BAT2* rs2280801, rs10885, rs1046089, rs2736158, rs1046080 and rs9366785 were all greater than 99%. All SNPs conformed to HWE (*p* > .001). The MAFs in our cohort were consistent with the online data for the Chinese Han population in the 1000 Genomes Project, indicating that our dataset is reliable. As shown in Table 1, we noted significant differences in the genotypic and allelic distribution for rs1046089 and rs9366785 between low responders and responders. Supplementary Table S3 showed the comparison of genotypic frequencies of six tag SNPs in different genetic models between two groups. According to the optimal genetic model for each variant, univariable and multivariable logistic regression analyses were performed and presented in Table 2. Compared with the rs1046089GG genotype, the GA + AA genotype was correlated with decreased risk of low responsiveness to influenza vaccines after adjusting for gender and age (*p* = 1.12E-03, OR = .562, 95%CI: .398–.795). *BAT2* rs9366785 GA + AA genotype was associated with a higher risk of low responsiveness to influenza vaccination compared with the GG genotype (*p* = .003, OR = 1.854, 95%CI: 1.229–2.799). All the statistical differences are robust to the Bonferroni correction (*p* < .05/6 = 8.33E-03).

**TABLE 1:** Comparison of genotypic and allelic frequencies of six tag SNPs in BAT2 between two groups.

SNP	Genotype/Allele	LRs (%)	Responders (%)	*p*	HWE- P	MAF	MAF of Chinese in database	Call rate (%)
rs2280801	CC	152(67.3)	232(64.1)	.556	.426	.189	.184	99.32
	CT	66(29.2)	120(33.1)					
	TT	8(3.5)	10(2.8)					
	C	370(81.9)	584(80.7)	.610				
	T	82(18.1)	140(19.3)					
rs10885	CC	190(84.1)	295(81.7)	.640^a^	.070	.095	.102	99.16
	CT	32(14.1)	61(16.9)					
	TT	4(1.8)	5(1.4)					
	C	412(91.2)	651(90.2)	.575				
	T	40(8.8)	71(9.8)					
rs1046089	GG	102(44.9)	113(31.1)	2.60E-04	.452	.403	.388	99.66
	GA	101(44.5)	174(47.9)					
	AA	24(10.6)	76(21.0)					
	G	305(67.2)	400(55.1)	3.80E-05				
	A	149(32.8)	326(44.9)					
rs2736158	GG	171(76.0)	284(78.5)	.757^a^	.627	.118	.121	99.16
	GC	51(22.7)	74(20.4)					
	CC	3(1.3)	4(1.1)					
	G	393(87.3)	642(88.7)	.489				
	C	57(12.7)	82(11.3)					
rs1046080	AA	207(92.0)	329(90.6)	.786^a^	.474	.046	.073	99.32
	AC	17(7.6)	33(9.1)					
	CC	1(.4)	1(.3)					
	A	431(95.8)	691(95.2)	.634				
	C	19(4.2)	35(4.8)					
rs9366785	GG	167(73.6)	308(84.4)	.004^a^	.542	.106	.107	100.00
	GA	56(24.7)	53(14.5)					
	AA	4(1.7)	4(1.1)					
	G	390(85.9)	669(91.6)	.002				
	A	64(14.1)	61(8.4)					

^a^ Fisher exact test. LR, low responder; SNP, single nucleotide polymorphism; HWE, Hardy-Weinberg equilibrium; MAF, minor allele frequency.

**TABLE 2:** Univariable and multivariable logistic regression analysis of six tag SNPs.

SNP	Genetic model	Genotype	Univariable		Multivariable	
			*p*	OR (95%CI)	*P* ^a^	OR (95%CI)^a^
rs2280801	Overdominant	CC + TT		1.00		1.00
		CT	.317	.832(.580–1.193)	.292	.823(.572–1.182)
rs10885	Overdominant	CC + TT		1.00		1.00
		CT	.377	.811(.510–1.291)	.354	.801(.502–1.280)
rs1046089	Dominant	GG		1.00		1.00
		GA + AA	7.41E-04	.554(.393–.781)	1.12E-03	.562(.398–.795)
rs2736158	Dominant	GG		1.00		1.00
		GC + CC	.489	1.150(.774–1.708)	.388	1.192(.800–1.776)
rs1046080	Overdominant	AA + CC		1.00		1.00
		AC	.517	.817(.444–1.505)	.553	.830(.448–1.536)
rs9366785	Dominant	GG		1.00		1.00
		GA + AA	1.46E-03	1.941(1.290–2.921)	.003	1.854(1.229–2.799)

^a^ Adjusted for age and gender. SNP, single nucleotide polymorphism; OR, odds ratio; CI, confidence interval.

**FIGURE 3:**
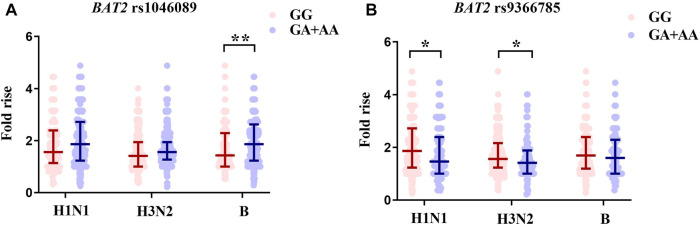
Comparison of antibody fold rises against different vaccine strains. **(A)** The comparison of antibody fold rises between rs1046089GG genotype and GA + AA genotype carriers. **(B)** The comparison of antibody fold rises between rs9366785GG genotype and GA + AA genotype carriers. Notes: Significant differences were marked as *p < .05, **p < .01.

For two SNPs with statistical differences identified in the association analysis, we further compared the antibody fold rises against three vaccine strains of different genotype carriers (Figure 3; Table 3). Compared with rs1046089GG carriers, GA + AA carriers had higher antibody fold rises to B (*p* = .004). The antibody fold rises of subjects with rs9366785 GA + AA genotype against H1N1 (*p* = .037) and H3N2 (*p* = .023) were significantly lower than those of subjects with GG genotype.

**TABLE 3:** Comparison of antibody fold rises against three vaccine strains of different genotype carriers.

Strain	rs1046089				rs9366785			
	GG	GA + AA	*p*	*p* ^a^	GG	GA + AA	*p*	*p* ^a^
H1N1	1.56(1.14–2.39)	1.86(1.23–2.72)	.056	.066	1.86(1.23–2.72)	1.46(1.00–2.39)	.021	.037
H3N2	1.41(1.00–1.94)	1.56(1.27–1.94)	.010	.069	1.56(1.23–2.16)	1.41(1.00–1.88)	.007	.023
B	1.43(1.00–2.29)	1.86(1.23–2.62)	.004	.004	1.69(1.19–2.39)	1.60(1.00–2.29)	.094	.155

The HI, titers before and after vaccination were transformed to their logarithms. The fold rises were described as the median (IQR).

^a^ *p* values were calculated using covariance analysis adjusted for gender and age.

#### Stratification analysis

Supplementary Table S4 presented the genotypic frequencies of six tag SNPs between low responders and responders in males and females. According to the optimal genetic model for each SNP in different gender groups, multivariable logistic regression analysis showed that compared with the rs9366785 GG + AA genotype, GA genotype was correlated with an increased risk of low responsiveness in males (*p* = 7.00E-04, OR = 3.280, 95%CI: 1.650–6.518) (Table 4). In females, we found that rs2280801CT genotype was statistically associated with a lower risk of low responsiveness to influenza vaccines compared with the CC + TT genotype (*p* = .008, OR = .515, 95%CI: .315–.844). And the immune response level of rs1046089 AA carriers was higher than that of GG + GA carriers (*p* = 1.25E-03, OR = .287, 95%CI: .135–.613) (Table 4). In Supplementary Tables S5, S6, we summarized and compared the genotypic frequencies of six tag SNPs between low responders and responders in different age groups. Multivariable logistic regression analysis showed that in the elderly group, rs9366785GA + AA genotype was significantly correlated with a higher risk of low responsiveness to influenza vaccines compared with the GG genotype (*p* = .004, OR = 3.792, 95%CI: 1.529-9.402) (Table 5), and the statistical difference is still robust to the Bonferroni correction.

**TABLE 4:** Multivariable logistic regression analysis of six tag SNPs in males and females.

SNP	Male				Female				
	Genetic model	Genotype	*P* ^a^	OR (95%CI)^a^	Genetic model	Genotype		*P* ^a^	OR (95%CI)^a^
rs2280801	Overdominant	CC + TT		1.00	Overdominant	CC + TT			1.00
		CT	.110	1.581(.901–2.775)		CT		.008	.515(.315–.844)
rs10885	Dominant	CC		1.00	Recessive	CC + CT			1.00
		CT + TT	.083	.531(.259–1.086)		TT		.468	1.825(.359–9.277)
rs1046089	Dominant	GG		1.00	Recessive	GG + GA			1.00
		GA + AA	.018	.505(.286–.890)		AA		1.25E-03	.287(.135–.613)
rs2736158	Dominant	GG		1.00	Dominant	GG			1.00
		GC + CC	.557	1.214(.636–2.318)		GC + CC		.491	1.196(.719–1.991)
rs1046080	Dominant	AA		1.00	Dominant	AA			1.00
		AC + CC	.643	1.249(.488–3.196)		AC + CC		.264	.630(.281–1.416)
rs9366785	Overdominant	GG + AA		1.00	Dominant	GG			1.00
		GA	7.00E-04	3.280(1.650–6.518)		GA + AA		.208	1.408(.827–2.399)

^a^ Adjusted for age. SNP, single nucleotide polymorphism; OR, odds ratio; CI, confidence interval.

**TABLE 5:** Multivariable logistic regression analysis of six tag SNPs in different age groups.

SNP	Infants (<5 years)				Children (5–17 years)			
	Genetic model	Genotype	*P* ^a^	OR (95%CI)^a^	Genetic model	Genotype	*P* ^a^	OR (95%CI)^a^
rs2280801	Recessive	CC + CT		1.00	Dominant	CC		1.00
		TT	.707	1.467(.198–10.859)		CT + TT	.152	2.469(.718–8.489)
rs10885	Dominant	CC		1.00	Dominant	CC		1.00
		CT + TT	.438	1.318(.656–2.647)		CT + TT	.336	.441(.083–2.336)
rs1046089	Dominant	GG		1.00	Recessive	GG + GA		1.00
		GA + AA	.084	.537(.265–1.088)		AA	.290	.395(.071–2.210)
rs2736158	Dominant	GG		1.00	Overdominant	GG + CC		1.00
		GC + CC	.434	1.384(.614–3.119)		GC	.779	1.197(.342–4.187)
rs1046080	Dominant	AA		1.00	Dominant	AA		1.00
		AC + CC	.368	.530(.133–2.111)		AC + CC	.999	—
rs9366785	Overdominant	GG + AA		1.00	Overdominant	GG + AA		1.00
		GA	.019	2.934(1.198–7.184)		GA	.559	1.748(.269–11.352)

SNP	Adults (18-64 years)				The elderly (≥65years)			
	Genetic Model	Genotype	*P* ^a^	OR (95%CI)^a^	Genetic Model	Genotype	*P* ^a^	OR (95%CI)^a^
rs2280801	Dominant	CC		1.00	Overdominant	CC+TT		1.00
		CT+TT	.051	.592(.350-1.002)		CT	.631	.820(.366-1.839)
rs10885	Recessive	CC+CT		1.00	Overdominant	CC+TT		1.00
		TT	.296	2.624(.429-16.045)		CT	.092	.411(.146-1.158)
rs1046089	Recessive	GG+GA		1.00	Dominant	GG		1.00
		AA	.014	.391(.185-.829)		GA+AA	.050	.462(.214-.998)
rs2736158	Overdominant	GG+CC		1.00	Recessive	GG+GC		1.00
		GC	.292	1.367(.764-2.446)		CC	.999	—
rs1046080	Recessive	AA+AC		1.00	Overdominant	AA+CC		1.00
		CC	1.000	—		AC	.274	3.633(.361-36.607)
rs9366785	Recessive	GG+GA		1.00	Dominant	GG		1.00
		AA	.137	5.642(.576-55.296)		GA+AA	.004	3.792(1.529-9.402)

^a^ Adjusted for gender. SNP, single nucleotide polymorphism; OR, odds ratio; CI, confidence interval.

#### Haplotype analysis

All pairwise *r* ^2^ estimates are lower than .05 (Supplementary Table S7), as expected from our tagging-based strategy that minimizes the association between SNPs. In Supplementary Table S8 and Table 6, we further investigated the effects of multi-SNP (haplotypic) in BAT2 on the immune response to influenza vaccination. The haplotype consisting of *BAT2* rs2280801-rs10885-rs1046089-rs2736158-rs1046080-rs9366785 CCAGAG was associated with a lower risk of low responsiveness to vaccines compared with haplotype CCGGAG (*p* < .001, OR = .37, 95%CI: .23–.58).

**TABLE 6:** Association between haplotypes and risk of low responsiveness to influenza vaccination.

Haplotype	SNPs						Frequency				OR (95%CI)^b^	*p* ^b^
	rs2280801	rs10885	rs1046089	rs2736158	rs1046080	rs9366785	Total	LRs	Responders			
1	C	C	G	G	A	G	.29	.36		.26	1.00	-
2	C	C	A	G	A	G	.21	.14		.26	.37 (.23–.58)	<.001
3	T	C	G	G	A	G	.11	.10		.11	.61 (.36–1.02)	.06
4	C	C	G	C	A	G	.07	.07		.06	.77 (.40–1.49)	.44
5	T	C	A	G	A	G	.05	.05		.06	.69 (.33–1.42)	.31
6	C	T	G	G	A	G	.05	.03		.06	.40 (.18–.91)	.03
7	C	C	G	G	A	A	.04	.06		.02	2.39 (1.01–5.68)	.05
8	C	T	A	G	A	G	.04	.04		.03	.91 (.40–2.07)	.83
9	C	C	A	C	A	G	.04	.03		.04	.76 (.32–1.84)	.55
10	C	C	G	G	C	G	.02	.01		.02	.80 (.29–2.23)	.67
11	T	C	A	G	A	A	.02	.02		.02	.80 (.29–2.21)	.67
12	C	C	A	G	C	G	.02	.02		.02	.59 (.17–2.00)	.40
Rare^a^	-	-	-	-	-	-	-	-		-	-	-

^a^ Rare: haplotypes with frequencies <.01.

^b^ Adjusted for age and gender. SNP, single nucleotide polymorphism; LR, low responder; OR, odds ratio; CI, confidence interval.

## Discussion

Our data demonstrate the first evidence that SNPs in BAT2 may be associated with the immune response to influenza vaccination in the Chinese Han population. We found that *BAT2* rs1046089 and rs9366785 were statistically correlated with the responsiveness to influenza vaccines both in qualitative analysis and quantitative analysis. *BAT2* rs1046089 is a missense variant that may result in a change of Arginine to Histidine from the change of G to A. The A allele is a protective factor for low antibody response. Thus, we hypothesized that the change from Arginine to Histidine in this locus may upregulate the immune responsiveness*. BAT2* rs9366785 is another significant missense SNP identified in our research. Alteration of allele G to A changes the amino acid from Glycine to Serine. Based on the risk effect of rs9366785 A allele, we speculated that the amino acid change in this position may lead to a poorer immune response to vaccination. Both rs1046089 and rs9366785 were predicted as splicing variants by SNPinfo. Diakite et al. found that the GG genotype of *BAT2* rs1046089 was a protective factor against severe malaria in the Gambian population (Diakite et al., 2009). Besides, no other studies have investigated these two variants. Based on the MAF and OR values of these two significant SNPs identified in our research, our sample size is sufficient with a statistical power of .80 (Supplementary Table S9).

A previous study speculated that the immunological effect of BAT2 may due to the linkage-disequilibrium between BAT2 and loci in the region of HLA class I, HLA class II and TNF (Fugger et al., 1990). To determine whether the role of BAT2 in the immune responsiveness to vaccines is independent, we summarized all the variants that were in LD (*r* ^2^ ≥ .8) with these six tag SNPs based on the HaploReg V4.1 online software. As shown in Supplementary Table S10, since we found that rs9366785 was not in LD with any other variant, and this tag SNP was also one of the most significant variants identified in our research, we further speculated that the effects of BAT2 on the immune system might be independent.

When stratified by gender, our study showed that the effects of each SNP differed between males and females. In our study, we only found weaker association evidence for gender difference with immune response. However, numerous studies have confirmed the superiority of women in the immune responsiveness to influenza vaccines (Furman et al., 2014; Giefing-Kröll et al., 2015; Klein & Flanagan, 2016). Engler et al. reported that women of all ages had been identified with higher geometric mean titers (GMTs) than men after vaccination, whatever the dose or strain (Engler et al., 2008). American researchers found that compared with adult males, adult females developed higher vaccine-induced immune responses (Potluri et al., 2019). In light of this phenomenon, some researchers believe that the reason why women’s responsiveness is higher than that of men can be attributed to the difference in the sex steroid hormone levels (Giefing-Kröll et al., 2015; Klein and Flanagan, 2016). In our study, according to the results of stratification analysis, we found that the protective effect of rs1046089 GA + AA genotype was diminished in males, while the risk effect of rs9366785GA + AA genotype was diminished in females. These phenomena suggested the interaction between SNPs and gender in the responsiveness to influenza vaccine. The risk genotypes were synergistic with the male but antagonistic with the female. After stratified by gender, the protective effect of rs2280801 CT genotype was only found in females and the difference was statistically significant even after the Bonferroni correction. We speculated that the protective effect of this variant may be partially diminished due to the poorer immune response to vaccines in males. *BAT2* rs2280801 is a missense and splicing variant that may result in a change of Proline to Leucine from the change of C to T. A previous study of African Americans reported that rs2280801 was correlated with the viral load set point of human immunodeficiency virus type 1(Pelak et al., 2010), indicating the potential role of this variant in the immune system. Since we found no association between rs2280801 and the immune responses before the stratification analysis, future work on this variant should be carried out in a larger scale cohort for further validation.

Age is also an important factor that may influence the antibody responses to vaccines. According to the data of 2015–2016 influenza epidemic season in Poland, a study reported that the GMT level and seroprotection rate of children aged 0–4 and the elderly over 65 were both lower, suggesting the susceptibility of these two age groups to influenza (Kowalczyk et al., 2019). The immune system of infants is not fully developed, their magnitude and activity of antigen-presenting cells, immune cells and cytokines are significantly lower than adults and older children (X. Zhang et al., 2017). Unlike infants, the underlying cause of the low responsiveness to vaccines in the elderly is age-dependent decrease in immunological competence, often referred to as ''immunosenescence'' (Sadighi Akha, 2018). After stratified by age, only one significant association was found in old people aged over 65 years. Compare with the GG genotype of rs9366785, the risk ratio of GA + AA genotype in the elderly group was higher than that of the whole age group (3.792 vs1.854). This suggested that there may be a synergistic effect between older age and rs9366785GA + AA genotype. Therefore, for old people with rs9366785 A allele, a booster vaccination before the flu season may be necessary.

In our study, no difference in rs10885 and rs2736158 distribution was found between low responders and responders. Previous studies have shown that these two SNPs may be candidate targets for some immune-mediated diseases. Walsh et al. found that rs2736158 C>G substitution changes an alanine residue to a glycine residue, and is found at a frequency of 12% in controls where it acts as a protective allele decreasing the risk of squamous cell carcinoma in African Americans (*p* = 1.82E-03) (Walsh et al., 2013). A study conducted in the Han Chinese population reported that rs2736157 AG genotype was associated with increased risk susceptibility for AQP4^+^ neuromyelitis optica spectrum disorder (NMOSD) (*p* = .03) (J. Zhang et al., 2021). As presented in Supplementary Table S10, rs2736157 was in strong LD with rs10885 (*r* ^2^ = .99), indicating the potential association between rs10885 and NMOSD. However, there is still no strong evidence to support the impacts of these two variants on vaccine immunity.

Haplotype analysis demonstrated the cumulative effect of various SNPs. Compared with the most common haplotype CCGGAG, the substitution of rs1046089 A allele, rs2280801T allele, and rs10885 T allele in the haplotype all showed protective effects. Among them, rs1046089 A allele had the highest impact and weight. The substitution of rs9366785 A allele in the haplotype was infrequent, and the risk effect of it was diminished by the collaboration of rs1046089 A allele and rs2280801T allele. In summary, according to the effect and haplotype frequency of six SNPs included in this study, the variation of rs1046089 showed the greatest impact on vaccine immunity in the Chinese population.

To enhance the power of our analysis, a series of quality controls were processed in the stage of subject selection and statistical analysis. Nevertheless, this study still has some limitations. Firstly, in the multivariable analysis, we only included two factors (age and gender) for adjustment. Secondly, due to the limitation of genotyping technology, except for rs9366785, we failed to determine the genotypes of all subjects, which may lead to certain biases. Thirdly, the immune effects of these variants have not been validated in animal models based on population studies. Finally, we only found evidence of an association between BAT2 and the immune system but failed to reveal its specific function. The role of HLA class III regions in immunity needs to be further explored.

In conclusion, our study identified two variants, rs1046089 and rs9366785, that were associated with the immune response to influenza vaccination in the Chinese Han population. The identification of population-specific functional variants will assist the design of new influenza vaccines with superior effectiveness, improve the individualized influenza vaccination program and provide evidence for further exploration in the HLA class III region.

## Data Availability

The raw data that support the findings of this study are available from the corresponding author, upon reasonable request.
