# Supplementary material for: Genetic variants in BAT2 are associated with immune responsiveness to influenza vaccination
Source: Front Genet. 2023 Feb 9;14:1059447. doi: 10.3389/fgene.2023.1059447 (PMC9951381; doi:10.3389/fgene.2023.1059447)
Supplement: Supplementary file 1 [file Table1.pdf]

Table S1. Vaccine strains recommended by WHO for the northern hemisphere from 2009-2019

Flu season	H1N1	H3N2	BV/BY
2009-2010	A/Brisbane/59/2007	A/Brisbane/10/2007	B/Brisbane/60/2008(BV)
2010-2011	A/California/7/2009	A/Perth/16/2009	B/Brisbane/60/2008(BV)
2011-2012	A/California/7/2009	A/Perth/16/2009	B/Brisbane/60/2008(BV)
2012-2013	A/California/7/2009	A/Victoria/361/2011	B/Wisconsin/1/2010(BY)
2013-2014	A/California/7/2009	A/Texas/50/2012	B/Massachusetts/2/2012(BY)
2014-2015	A/California/7/2009	A/Texas/50/2012	B/Massachusetts/2/2012(BY)
2015-2016	A/California/7/2009	A/Switzerland/9715293/2013	B/Phuket/3073/2013(BY)
2016-2017	A/California/7/2009	A/Hong Kong/4801/2014	B/Brisbane/60/2008(BV)
2017-2018	A/Michigan/45/2015	A/Hong Kong/4801/2014	B/Brisbane/60/2008(BV)
2018-2019	A/Michigan/45/2015	A/Singapore/INFIMH-16-0019/2016	B/Colorado/06/2017 (BV)
2019-2020	A/Brisbane/02/2018	A/Kansas/14/2017	B/Colorado/06/2017 (BV)

Table S2. Comparison of characteristics of subjects between low responders and responders

Variants	LRs (%) (n=227)	Responders (%) (n=365)	*P*
Gender			
Male	91(40.1)	142(38.9)	0.774
Female	136(59.9)	223(61.1)	
Age			
<5	54(23.8)	84(23.0)	0.102
5-17	16(7.1)	45(12.3)	
18-64	107(47.1)	176(48.2)	
≥65	50(22.0)	60(16.5)	

LR, Low responder.

Table S3. Comparison of genotypic frequencies of 6 tag SNPs in different genetic models between two groups

SNP	Genetic Model	Genotype	LRs (%)	Responders (%)	*P*
rs2280801	Dominant	CC	152(67.3)	232(64.1)	0.432
		CT+TT	74(32.7)	130(35.9)	
	Recessive	CC+CT	218(96.5)	352(97.2)	0.595
		TT	8(3.5)	10(2.8)	
	Overdominant	CC+TT	160(70.8)	242(66.9)	0.317
		CT	66(29.2)	120(33.1)	
rs10885	Dominant	CC	190(84.1)	295(81.7)	0.464
		CT+TT	36(15.9)	66(18.3)	
	Recessive	CC+CT	222(98.2)	356(98.6)	0.739[a]
		TT	4(1.8)	5(1.4)	
	Overdominant	CC+TT	194(85.8)	300(83.1)	0.377
		CT	32(14.2)	61(16.9)	
rs1046089	Dominant	GG	102(44.9)	113(31.1)	6.99E-04
		GA+AA	125(55.1)	250(68.9)	
	Recessive	GG+GA	203(89.4)	287(79.1)	1.10E-03
		AA	24(10.6)	76(20.9)	
	Overdominant	GG+AA	126(55.5)	189(52.1)	0.415
		GA	101(44.5)	174(47.9)	
rs2736158	Dominant	GG	171(76.0)	284(78.5)	0.489
		GC+CC	54(24.0)	78(21.5)	
	Recessive	GG+GC	222(98.7)	358(98.9)	1.000[a]
		CC	3(1.3)	4(1.1)	
	Overdominant	GG+CC	174(77.3)	288(79.6)	0.522
		GC	51(22.7)	74(20.4)	
rs1046080	Dominant	AA	207(92.0)	329(90.6)	0.571
		AC+CC	18(8.0)	34(9.4)	
	Recessive	AA+AC	224(99.6)	362(99.7)	1.000[a]
		CC	1(0.4)	1(0.3)	
	Overdominant	AA+CC	208(92.4)	330(90.9)	0.517
		AC	17(7.6)	33(9.1)	
rs9366785	Dominant	GG	167(73.6)	308(84.4)	1.31E-03
		GA+AA	60(26.4)	57(15.6)	
	Recessive	GG+GA	223(98.2)	361(98.9)	0.490[a]
		AA	4(1.8)	4(1.1)	
	Overdominant	GG+AA	171(75.3)	312(85.5)	1.95E-03
		GA	56(24.7)	53(14.5)	

[a] Fisher exact test. LR, Low responder; SNP, single nucleotide polymorphism.

Table S4. Comparison of genotypic frequencies of 6 tag SNPs between two groups in males and females

SNP	Genetic Model	Genotype	Male			Female		
			LRs (%)	Responders (%)	*P*	LRs (%)	Responders (%)	*P*
rs2280801	Additive	CC	53(58.2)	95(67.9)	0.322[a]	99(73.3)	137(61.7)	0.029[a]
		CT	36(39.6)	42(30.0)		30(22.2)	78(35.1)	
		TT	2(2.2)	3(2.1)		6(4.5)	7(3.2)	
	Dominant	CC	53(58.2)	95(67.9)	0.137	99(73.3)	137(61.7)	0.024
		CT+TT	38(41.8)	45(32.1)		36(26.7)	85(38.3)	
	Recessive	CC+CT	89(97.8)	137(97.9)	1.000[a]	129(95.5)	215(96.8)	0.568[a]
		TT	2(2.2)	3(2.1)		6(4.5)	7(3.2)	
	Overdominant	CC+TT	55(60.4)	98(70.0)	0.133	105(77.8)	144(64.9)	0.010
		CT	36(39.6)	42(30.0)		30(22.2)	78(35.1)	
rs10885	Additive	CC	78(85.7)	108(77.2)	0.223[a]	112(83.0)	187(84.6)	0.817[a]
		CT	12(13.2)	30(21.4)		20(14.8)	31(14.0)	
		TT	1(1.1)	2(1.4)		3(2.2)	3(1.4)	
	Dominant	CC	78(85.7)	108(77.2)	0.108	112(83.0)	187(84.6)	0.680
		CT+TT	13(14.3)	32(22.8)		23(17.0)	34(15.4)	
	Recessive	CC+CT	90(98.9)	138(98.6)	1.000[a]	132(97.8)	218(98.6)	0.677[a]
		TT	1(1.1)	2(1.4)		3(2.2)	3(1.4)	
	Overdominant	CC+TT	79(86.8)	110(78.6)	0.113	115(85.2)	190(86.0)	0.837
		CT	12(13.2)	30(21.4)		20(14.8)	31(14.0)	
rs1046089	Additive	GG	41(45.1)	41(29.3)	0.046	61(44.9)	72(32.3)	1.54E-03
		GA	35(38.4)	66(47.1)		66(48.5)	108(48.4)	
		AA	15(16.5)	33(23.6)		9(6.6)	43(19.3)	
	Dominant	GG	41(45.1)	41(29.3)	0.014	61(44.9)	72(32.3)	0.017
		GA+AA	50(54.9)	99(70.7)		75(55.1)	151(67.7)	
	Recessive	GG+GA	76(83.5)	107(76.4)	0.195	127(93.4)	180(80.7)	9.41E -04
		AA	15(16.5)	33(23.6)		9(6.6)	43(19.3)	
	Overdominant	GG+AA	56(61.6)	74(52.9)	0.194	70(51.5)	115(51.6)	0.985
		GA	35(38.4)	66(47.1)		66(48.5)	108(48.4)	
rs2736158	Additive	GG	69(76.7)	110(78.6)	0.937[a]	102(75.5)	174(78.4)	0.859[a]
		GC	20(22.2)	29(20.7)		31(23.0)	45(20.3)	
		CC	1(1.1)	1(0.7)		2(1.5)	3(1.3)	
	Dominant	GG	69(76.7)	110(78.6)	0.734	102(75.5)	174(78.4)	0.537
		GC+CC	21(23.3)	30(21.4)		33(24.5)	48(21.6)	
	Recessive	GG+GC	89(98.9)	139(99.3)	1.000[a]	133(98.5)	219(98.7)	1.000[a]
		CC	1(1.1)	1(0.7)		2(1.5)	3(1.3)	
	Overdominant	GG+CC	70(77.8)	111(79.3)	0.785	104(77.0)	177(79.7)	0.547
		GC	20(22.2)	29(20.7)		31(23.0)	45(20.3)	

[a] Fisher exact test. LR, low responder; SNP, single nucleotide polymorphism.

Table S4. Comparison of genotypic frequencies of 6 tag SNPs between two groups in males and females (Continue)

SNP	Genetic Model	Genotype	Male			Female		
			LRs (%)	Responders (%)	*P*	LRs (%)	Responders (%)	*P*
rs1046080	Additive	AA	81(90.0)	129(92.1)	0.515[a]	126(93.3)	200(89.7)	0.530[a]
		AC	8(8.9)	11(7.9)		9(6.7)	22(9.9)	
		CC	1(1.1)	0(0.0)		0(0.0)	1(0.4)	
	Dominant	AA	81(90.0)	129(92.1)	0.574	126(93.3)	200(89.7)	0.241
		AC+CC	9(10.0)	11(7.9)		9(6.7)	23(10.3)	
	Recessive	AA+AC	89(98.9)	140(100.0)	0.391[a]	135(100.0)	222(99.6)	1.000[a]
		CC	1(1.1)	0(0.0)		0(0.0)	1(0.4)	
	Overdominant	AA+CC	82(91.1)	129(92.1)	0.781	126(93.3)	201(90.1)	0.297
		AC	8(8.9)	11(7.9)		9(6.7)	22(9.9)	
rs9366785	Additive	GG	63(69.2)	124(87.3)	5.88E-04[a]	104(76.5)	184(82.5)	0.269[a]
		GA	28(30.8)	17(12.0)		28(20.6)	36(16.1)	
		AA	0(0.0)	1(0.7)		4(2.9)	3(1.4)	
	Dominant	GG	63(69.2)	124(87.3)	7.12E-04	104(76.5)	184(82.5)	0.163
		GA+AA	28(30.8)	18(12.7)		32(23.5)	39(17.5)	
	Recessive	GG+GA	91(100.0)	141(99.3)	1.000[a]	132(97.1)	220(98.6)	0.433[a]
		AA	0(0.0)	1(0.7)		4(2.9)	3(1.4)	
	Overdominant	GG+AA	63(69.2)	125(88.0)	3.91E-04	108(79.4)	187(83.9)	0.286
		GA	28(30.8)	17(12.0)		28(20.6)	36(16.1)	

[a] Fisher exact test. LR, low responder; SNP, single nucleotide polymorphism.

Table S5. Comparison of genotypic frequencies of 6 tag SNPs between two groups in infants and children

SNP	Genetic Model	Genotype	Infants (<5 years)			Children (5-17 years)		
			LRs (%)	Responders (%)	*P*	LRs (%)	Responders (%)	*P*
rs2280801	Additive	CC	34(63.0)	55(65.5)	0.846[a]	9(56.2)	32(74.4)	0.212[a]
		CT	18(33.3)	27(32.1)		7(43.8)	11(25.6)	
		TT	2(3.7)	2(2.4)		0(0.0)	0(0.0)	
	Dominant	CC	34(63.0)	55(65.5)	0.763	9(56.2)	32(74.4)	0.212[a]
		CT+TT	20(37.0)	29(34.5)		7(43.8)	11(25.6)	
	Recessive	CC+CT	52(96.3)	82(97.6)	0.644[a]	16(100.0)	43(100.0)	—
		TT	2(3.7)	2(2.4)		0(0.0)	0(0.0)	
	Overdominant	CC+TT	36(66.7)	57(67.9)	0.884	9(56.2)	32(74.4)	0.212[a]
		CT	18(33.3)	27(32.1)		7(43.8)	11(25.6)	
rs10885	Additive	CC	47(87.0)	69(84.2)	0.883[a]	14(87.5)	34(77.3)	0.787[a]
		CT	7(13.0)	12(14.6)		2(12.5)	9(20.4)	
		TT	0(0.0)	1(1.2)		0(0.0)	1(2.3)	
	Dominant	CC	47(87.0)	69(84.2)	0.641	14(87.5)	34(77.3)	0.486[a]
		CT+TT	7(13.0)	13(15.8)		2(12.5)	10(22.7)	
	Recessive	CC+CT	54(100.0)	81(98.8)	1.000[a]	16(100.0)	43(97.7)	1.000[a]
		TT	0(0.0)	1(1.2)		0(0.0)	1(2.3)	
	Overdominant	CC+TT	47(87.0)	70(85.4)	0.783	14(87.5)	35(79.6)	0.710[a]
		CT	7(13.0)	12(14.6)		2(12.5)	9(20.4)	
rs1046089	Additive	GG	26(48.1)	28(33.7)	0.235	6(37.5)	14(31.8)	0.567[a]
		GA	21(38.9)	40(48.2)		8(50.0)	18(40.9)	
		AA	7(13.0)	15(18.1)		2(12.5)	12(27.3)	
	Dominant	GG	26(48.1)	28(33.7)	0.092	6(37.5)	14(31.8)	0.680
		GA+AA	28(51.9)	55(66.3)		10(62.5)	30(68.2)	
	Recessive	GG+GA	47(87.0)	68(81.9)	0.426	14(87.5)	32(72.7)	0.314[a]
		AA	7(13.0)	15(18.1)		2(12.5)	12(27.3)	
	Overdominant	GG+AA	33(61.1)	43(51.8)	0.284	8(50.0)	26(59.1)	0.530
		GA	21(38.9)	40(48.2)		8(50.0)	18(40.9)	
rs2736158	Additive	GG	40(74.1)	66(79.5)	0.771[a]	11(68.8)	31(70.4)	1.000[a]
		GC	13(24.1)	16(19.3)		5(31.2)	12(27.3)	
		CC	1(1.8)	1(1.2)		0(0.0)	1(2.3)	
	Dominant	GG	40(74.1)	66(79.5)	0.457	11(68.8)	31(70.4)	1.000[a]
		GC+CC	14(25.9)	17(20.5)		5(31.2)	13(29.6)	
	Recessive	GG+GC	53(98.2)	82(98.8)	1.000[a]	16(100.0)	43(97.7)	1.000[a]
		CC	1(1.8)	1(1.2)		0(0.0)	1(2.3)	
	Overdominant	GG+CC	41(75.9)	67(80.7)	0.502	11(68.8)	32(72.7)	0.756[a]
		GC	13(24.1)	16(19.3)		5(31.2)	12(27.3)	

[a] Fisher exact test. LR, low responder; SNP, single nucleotide polymorphism.

Table S5. Comparison of genotypic frequencies of 6 tag SNPs between two groups in infants and children (Continue)

SNP	Genetic Model	Genotype	Infants (<5 years)			Children (5-17 years)		
			LRs (%)	Responders (%)	*P*	LRs (%)	Responders (%)	*P*
rs1046080	Additive	AA	51(94.4)	76(90.5)	0.528[a]	16(100.0)	41(93.2)	0.558[a]
		AC	3(5.6)	8(9.5)		0(0.0)	3(6.8)	
		CC	0(0.0)	0(0.0)		0(0.0)	0(0.0)	
	Dominant	AA	51(94.4)	76(90.5)	0.528[a]	16(100.0)	41(93.2)	0.558[a]
		AC+CC	3(5.6)	8(9.5)		0(0.0)	3(6.8)	
	Recessive	AA+AC	54(100.0)	84(100.0)	—	16(100.0)	44(100.0)	—
		CC	0(0.0)	0(0.0)		0(0.0)	0(0.0)	
	Overdominant	AA+CC	51(94.4)	76(90.5)	0.528[a]	16(100.0)	41(93.2)	0.558[a]
		AC	3(5.6)	8(9.5)		0(0.0)	3(6.8)	
rs9366785	Additive	GG	39(72.2)	72(85.7)	0.025[a]	14(87.5)	41(91.1)	0.648[a]
		GA	15(27.8)	10(11.9)		2(12.5)	4(8.9)	
		AA	0(0.0)	2(2.4)		0(0.0)	0(0.0)	
	Dominant	GG	39(72.2)	72(85.7)	0.051	14(87.5)	41(91.1)	0.648[a]
		GA+AA	15(27.8)	12(14.3)		2(12.5)	4(8.9)	
	Recessive	GG+GA	54(100.0)	82(97.6)	0.520[a]	16(100.0)	45(100.0)	—
		AA	0(0.0)	2(2.4)		0(0.0)	0(0.0)	
	Overdominant	GG+AA	39(72.2)	74(88.1)	0.018	14(87.5)	41(91.1)	0.648[a]
		GA	15(27.8)	10(11.9)		2(12.5)	4(8.9)	

[a] Fisher exact test. LR, low responder; SNP, single nucleotide polymorphism.

Table S6. Comparison of genotypic frequencies of 6 tag SNPs between two groups in adults and the elderly

SNP	Genetic Model	Genotype	Adults (18-64 years)			The elderly (≥65 years)		
			LRs (%)	Responders (%)	*P*	LRs (%)	Responders (%)	*P*
rs2280801	Additive	CC	78(72.9)	108(61.7)	0.142[a]	31(63.3)	37(61.7)	0.751[a]
		CT	26(24.3)	61(34.9)		15(30.6)	21(35.0)	
		TT	3(2.8)	6(3.4)		3(6.1)	2(3.3)	
	Dominant	CC	78(72.9)	108(61.7)	0.054	31(63.3)	37(61.7)	0.864
		CT+TT	29(27.1)	67(38.3)		18(36.7)	23(38.3)	
	Recessive	CC+CT	104(97.2)	169(96.6)	1.000[a]	46(93.9)	58(96.7)	0.656[a]
		TT	3(2.8)	6(3.4)		3(6.1)	2(3.3)	
	Overdominant	CC+TT	81(75.7)	114(65.1)	0.063	34(69.4)	39(65.0)	0.628
		CT	26(24.3)	61(34.9)		15(30.6)	21(35.0)	
rs10885	Additive	CC	86(81.1)	148(84.6)	0.502[a]	43(86.0)	44(73.3)	0.157[a]
		CT	17(16.1)	25(14.3)		6(12.0)	15(25.0)	
		TT	3(2.8)	2(1.1)		1(2.0)	1(1.7)	
	Dominant	CC	86(81.1)	148(84.6)	0.454	43(86.0)	44(73.3)	0.104
		CT+TT	20(18.9)	27(15.4)		7(14.0)	16(26.7)	
	Recessive	CC+CT	103(97.2)	173(98.9)	0.369[a]	49(98.0)	59(98.3)	1.000[a]
		TT	3(2.8)	2(1.1)		1(2.0)	1(1.7)	
	Overdominant	CC+TT	89(83.9)	150(85.7)	0.690	44(88.0)	45(75.0)	0.084
		CT	17(16.1)	25(14.3)		6(12.0)	15(25.0)	
rs1046089	Additive	GG	43(40.2)	50(28.4)	0.020	27(54.0)	21(35.0)	0.087
		GA	54(50.5)	90(51.1)		18(36.0)	26(43.3)	
		AA	10(9.3)	36(20.5)		5(10.0)	13(21.7)	
	Dominant	GG	43(40.2)	50(28.4)	0.041	27(54.0)	21(35.0)	0.045
		GA+AA	64(59.8)	126(71.6)		23(46.0)	39(65.0)	
	Recessive	GG+GA	97(90.7)	140(79.5)	0.014	45(90.0)	47(78.3)	0.100
		AA	10(9.3)	36(20.5)		5(10.0)	13(21.7)	
	Overdominant	GG+AA	53(49.5)	86(48.9)	0.913	32(64.0)	34(56.7)	0.434
		GA	54(50.5)	90(51.1)		18(36.0)	26(43.3)	
rs2736158	Additive	GG	79(75.2)	139(79.4)	0.390[a]	41(82.0)	48(80.0)	0.250[a]
		GC	26(24.8)	34(19.4)		7(14.0)	12(20.0)	
		CC	0(0.0)	2(1.2)		2(4.0)	0(0.0)	
	Dominant	GG	79(75.2)	139(79.4)	0.414	41(82.0)	48(80.0)	0.790
		GC+CC	26(24.8)	36(20.6)		9(18.0)	12(20.0)	
	Recessive	GG+GC	105(100.0)	173(98.8)	0.530[a]	48(96.0)	60(100.0)	0.204[a]
		CC	0(0.0)	2(1.2)		2(4.0)	0(0.0)	
	Overdominant	GG+CC	79(75.2)	141(80.6)	0.292	43(86.0)	48(80.0)	0.407
		GC	26(24.8)	34(19.4)		7(14.0)	12(20.0)	

[a] Fisher exact test. LR, low responder; SNP, single nucleotide polymorphism.

Table S6. Comparison of genotypic frequencies of 6 tag SNPs between two groups in adults and the elderly (Continue)

SNP	Genetic Model	Genotype	Adults (18-64 years)			The elderly (≥65 years)		
			LRs (%)	Responders (%)	*P*	LRs (%)	Responders (%)	*P*
rs1046080	Additive	AA	93(88.6)	154(88.0)	0.525[a]	47(94.0)	58(96.6)	0.328[a]
		AC	11(10.5)	21(12.0)		3(6.0)	1(1.7)	
		CC	1(0.9)	0(0.0)		0(0.0)	1(1.7)	
	Dominant	AA	93(88.6)	154(88.0)	0.886	47(94.0)	58(96.6)	0.657[a]
		AC+CC	12(11.4)	21(12.0)		3(6.0)	2(3.4)	
	Recessive	AA+AC	104(99.1)	175(100.0)	0.375[a]	50(100.0)	59(98.3)	1.000[a]
		CC	1(0.9)	0(0.0)		0(0.0)	1(1.7)	
	Overdominant	AA+CC	94(89.5)	154(88.0)	0.698	47(94.0)	59(98.3)	0.328[a]
		AC	11(10.5)	21(12.0)		3(6.0)	1(1.7)	
rs9366785	Additive	GG	84(78.5)	144(81.8)	0.303[a]	30(60.0)	51(85.0)	0.005[a]
		GA	20(18.7)	31(17.6)		19(38.0)	8(13.3)	
		AA	3(2.8)	1(0.6)		1(2.0)	1(1.7)	
	Dominant	GG	84(78.5)	144(81.8)	0.495	30(60.0)	51(85.0)	0.003
		GA+AA	23(21.5)	32(18.2)		20(40.0)	9(15.0)	
	Recessive	GG+GA	104(97.2)	175(99.4)	0.153[a]	49(98.0)	59(98.3)	1.000[a]
		AA	3(2.8)	1(0.6)		1(2.0)	1(1.7)	
	Overdominant	GG+AA	87(81.3)	145(82.4)	0.819	31(62.0)	52(86.7)	0.003
		GA	20(18.7)	31(17.6)		19(38.0)	8(13.3)	

[a] Fisher exact test. LR, low responder; SNP, single nucleotide polymorphism.

Table S7. The linkage disequilibrium coefficient between SNPs of BAT2

	rs2280801	rs10885	rs1046089	rs2736158	rs1046080	rs9366785
rs2280801	–	2.41E-02	1.49E-04	3.11E-02	1.11E-02	3.98E-03
rs10885	–	–	1.52E-05	1.39E-02	2.93E-03	1.94E-05
rs1046089	–	–	–	7.06E-05	3.25E-03	1.46E-04
rs2736158	–	–	–	–	6.35E-03	8.10E-05
rs1046080	–	–	–	–	–	5.20E-04
rs9366785	–	–	–	–	–	–

Values on the right of "–" are r^2. SNP, single nucleotide polymorphism.

Table S8. Frequencies of haplotypes of BAT2 among groups

Haplotype	rs2280801	rs10885	rs1046089	rs2736158	rs1046080	rs9366785	Total	LRs	Rs	Cumulative frequency
1	C	C	G	G	A	G	0.29	0.36	0.26	0.29
2	C	C	A	G	A	G	0.21	0.14	0.26	0.51
3	T	C	G	G	A	G	0.11	0.10	0.11	0.61
4	C	C	G	C	A	G	0.07	0.07	0.06	0.68
5	T	C	A	G	A	G	0.05	0.05	0.06	0.73
6	C	T	G	G	A	G	0.05	0.03	0.06	0.78
7	C	C	G	G	A	A	0.04	0.06	0.02	0.82
8	C	T	A	G	A	G	0.04	0.04	0.03	0.86
9	C	C	A	C	A	G	0.04	0.03	0.04	0.89
10	C	C	G	G	C	G	0.02	0.01	0.02	0.92
11	T	C	A	G	A	A	0.02	0.02	0.02	0.94
12	C	C	A	G	C	G	0.02	0.02	0.02	0.95
13	C	T	G	G	A	A	0.01	0.01	0.01	0.96
14	C	C	A	C	A	A	0.01	0.01	0.01	0.97
15	C	C	A	G	A	A	0.01	0.01	0.01	0.98
16	T	C	G	G	A	A	0.01	0.01	0.01	0.99
17	C	C	G	C	A	A	0.01	0.01	0.01	0.99
18	C	C	A	G	C	A	0.01	–	0.01	1.00
19	C	C	G	G	C	A	0.00	0.00	–	1.00
20	T	C	G	G	C	A	0.00	0.00	–	1.00
21	C	T	G	G	C	G	0.00	0.00	0.00	1.00

LR, low responder; R, responder.

Table S9. Calculation of minimum sample size in case and control group

SNPs	Genetic Model	OR	MAF	Controls per case	Population risk	Power	α	No. of cases	No. of controls
rs1046089	Dominant	0.562	0.403	365/227=1.61	0.15	0.80	0.05	160	258
rs9366785	Dominant	1.854	0.106	365/227=1.61	0.15	0.80	0.05	185	298

SNP, single nucleotide polymorphism; MAF, minor allele frequency; OR, odds ratio.

Table S10. Variants in linkage disequilibrium with 6 tag SNPs of BAT2

tag SNPs of BAT2	Chromosome	Position	r^2	D’	Variants	Gene
rs2280801	6	31605150	0.85	0.93	rs9378200	10kb 5' of AIF1
	6	31606269	0.8	0.95	rs6920486	8.9kb 5' of AIF1
	6	31606748	0.85	0.95	rs28895016	8.4kb 5' of AIF1
	6	31607499	0.82	0.95	rs9348876	7.7kb 5' of AIF1
	6	31607848	0.82	0.95	rs28732148	7.3kb 5' of AIF1
	6	31615447	0.9	0.95	rs28732150	AIF1
	6	31639897	0.88	0.95	rs28732154	BAG6
	6	31643285	0.97	0.98	rs9391731	BAG6
	6	31644799	0.95	0.98	rs12055599	BAG6
	6	31650946	0.97	0.98	rs9380266	BAG6
	6	31655039	0.97	0.98	rs9404941	APOM
	6	31660439	0.95	0.98	rs28732157	C6orf47
	6	31662541	0.95	0.98	rs28732158	GPANK1
	6	31675745	0.97	0.98	rs9378164	938bp 3' of LY6G5C
	6	31701518	0.93	0.96	rs2295663	ABHD16A
rs10885	6	31614248	0.87	0.98	rs3132451	935bp 5' of AIF1
	6	31619306	0.98	0.99	rs3130621	1.4kb 5' of BAT2
	6	31621487	0.98	1	rs3115665	BAT2
	6	31624031	1	1	rs3130070	BAT2
	6	31624747	1	1	rs3130622	BAT2
	6	31624859	1	1	rs3115664	BAT2
	6	31629923	0.92	0.98	rs3130623	BAT2
	6	31630098	0.99	1	rs3130624	BAT2
	6	31630712	0.99	1	rs3130626	BAT2
	6	31633043	0.99	1	rs2736157	BAT2
	6	31633074	0.99	1	rs3130627	BAT2
	6	31634066	0.98	0.99	rs3115663	BAT2
	6	31635993	1	1	rs11229	BAT2
	6	31638621	0.99	1	rs3130046	406bp 3' of BAG6
	6	31638900	0.87	1	rs199920659	127bp 3' of BAG6
	6	31606901	0.99	1	rs5875328	BAG6
	6	31641495	0.95	0.98	rs3130628	BAG6
	6	31642702	0.92	0.96	rs3130047	BAG6
	6	31651799	0.9	0.95	rs3117583	BAG6
	6	31663191	0.88	0.94	rs3117580	GPANK1
	6	31664357	0.87	0.94	rs3130618	GPANK1
	6	31665719	0.88	0.94	rs3117579	GPANK1
rs1046089	6	31629976	0.88	0.99	rs2242660	BAT2
	6	31633235	0.99	1	rs2844466	BAT2
	6	31633245	0.91	1	rs2844465	BAT2
	6	31633736	0.91	1	rs2242659	BAT2

Table S10. Variants in linkage disequilibrium with 6 tag SNPs of BAT2 (Continue)

tag SNPs of BAT2	Chromosome	Position	r^2	D'	Variants	Gene
rs1046089	6	31650180	0.85	0.96	rs805302	BAG6
	6	31650344	0.86	0.97	rs805301	BAG6
	6	31652243	0.86	0.97	rs813115	BAG6
	6	31666416	0.84	0.97	rs805257	CSNK2B
	6	31672741	0.84	0.97	rs1266076	LY6G5B
rs2736158	6	31617915	0.91	1	rs2857696	893bp 3' of AIF1
	6	31628898	0.91	1	rs2736165	BAT2
	6	31629931	0.91	1	rs2736163	BAT2
	6	31630516	1	1	rs2736161	BAT2
	6	31637390	1	1	rs2255741	BAT2
	6	31637501	0.9	1	rs1046756	BAT2
	6	31650790	0.91	1	rs805300	BAG6
	6	31653318	0.91	1	rs805298	APOM
	6	31654266	0.91	1	rs1266078	APOM
	6	31656096	1	1	rs805264	APOM
	6	31657764	0.91	1	rs707921	APOM
	6	31660336	0.9	1	rs805263	C6orf47
	6	31663600	0.91	1	rs805259	GPANK1
	6	31665775	1	1	rs805258	GPANK1
	6	31670401	1	1	rs805268	LY6G5B
	6	31671980	0.91	1	rs805267	LY6G5B
	6	31672475	0.91	1	rs805266	LY6G5B
	6	31673609	1	1	rs805272	LY6G5B
	6	31676313	1	1	rs805270	370bp 3' of LY6G5C
	6	31676505	0.91	1	rs805269	178bp 3' of LY6G5C
	6	31679876	0.81	0.9	rs805291	LY6G5C
	6	31679888	0.81	0.9	rs2736199	LY6G5C
	6	31690147	0.91	1	rs805283	BAT5
	6	31694492	0.91	1	rs805279	BAT5
	6	31696467	0.91	1	rs805275	BAT5
	6	31667202	0.9	1	rs1266072	BAT5
	6	31707520	0.91	1	rs805295	LY6G6F
	6	31717067	0.81	1	rs9461717	LY6G6F
	6	31745040	0.81	1	rs2075787	MSH5
	6	31746046	0.81	1	rs12177823	MSH5
	6	31754485	0.81	1	rs9296001	MSH5
	6	31755311	0.81	1	rs9469046	MSH5
	6	31760045	0.81	1	rs9461718	MSH5
	6	31764430	0.81	1	rs9461719	SAPCD1
	6	31770631	0.81	1	rs9461721	VWA7
	6	31777741	0.9	1	rs707927	VARS

Table S10. Variants in linkage disequilibrium with 6 tag SNPs of BAT2 (Continue)

tag SNPs of BAT2	Chromosome	Position	r^2	D’	Variants	Gene
rs1046080	6	31617223	1	1	rs2857597	201bp 3' of AIF1
	6	31623441	0.98	1	rs3130069	BAT2
	6	31633567	0.99	0.99	rs2272593	BAT2
	6	31659746	0.91	0.97	rs3130617	C6orf47
rs9366785	–	–	–	–	–	–
